# Supplementary material for: Artificial Intelligence Resolves Kinetic Pathways of Magnesium Binding to RNA
Source: J Chem Theory Comput. 2022 Jan 27;18(2):1202–12. doi: 10.1021/acs.jctc.1c00752 (PMC8830046; doi:10.1021/acs.jctc.1c00752)
Supplement: Supplementary file 1 — ct1c00752_si_001.pdf [file ct1c00752_si_001.pdf]

SUPPORTING INFORMATION

Artificial Intelligence Resolves Kinetic Pathways of Magnesium Binding to RNA

Jan Neumann[a] and Nadine Schwierz[b,*]

[a]Allianz Global Investors GmbH, Bockenheimer Landstrasse 42, 60323 Frankfurt am Main, Germany; [b]Department of Theoretical Biophysics, Max Planck Institute of Biophysics, 60438 Frankfurt am Main, Germany; *Corresponding author: nadine.schwierz@biophys.mpg.de

1 Convergence of the free energy profiles

The one-dimensional free energy profile as a function of the distance between Mg^{2+} and the phosphate oxygen O1P was calculated from umbrella sampling. To ensure convergence, each window was simulated for 100 ns and the results were divided into 8 blocks (Figure S1A). Each block yields identical results and agrees with the 100 ns simulation. Further insight into the quality of the umbrella sampling method is provided by the evenly spaced and overlapping histograms (Figure S1B).

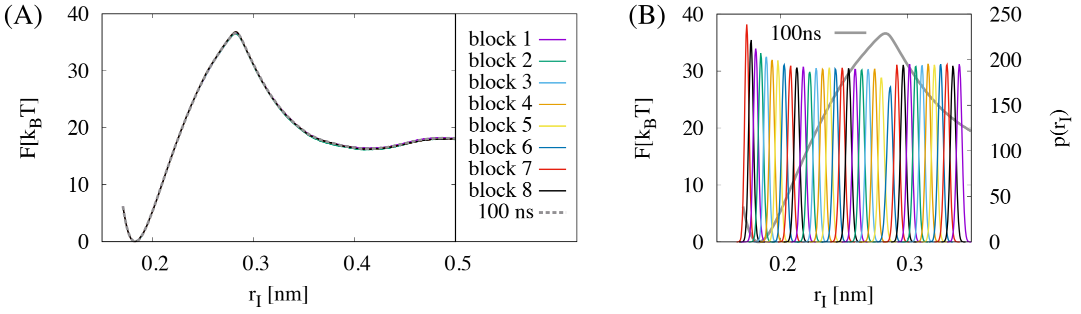

Figure S1: Convergence of the free energy profile. (A) Results from block averaging with 8 x 10.5 ns blocks. (B) Histograms from umbrella sampling.

2 Free energy landscape

The two-dimensional free energy landscape as a function of the distance between Mg^{2+} and the phosphate oxygen O1P is calculated from umbrella sampling. Without further restraints, the system has three stable states (Figure S2). State A corresponds to the inner-sphere conformation in which Mg^{2+} is coordinated by the O1P atom and five additional water molecules. State B corresponds to the outer-sphere conformation in which the O1P atom is replaced by the selected water molecule W_{ex} for which the biasing umbrella potential is applied. State C corresponds to conformations in which O1P is replaced by any other of the $N-6$ water molecules (where N is the number of water molecules in the simulations box and 6 is the coordination number). In this two-dimensional projection, transitions between state A and state C involve additional water molecules that are hidden in the selected two-dimensional projection. Here, an $N-6$ dimensional representation would be required to capture all relevant coordinates. However, since all outer-sphere states are identical since the water molecules are indistinguishable, it is sufficient to show only the exchanges between state A and state B in the two-dimensional projection. This is achieved by applying an additional biasing potential on the coordination number n_1. Hereby, the coordination number is calculated from the switching function

$$s(r) = \frac{1-(r/r_0)^{24}}{1-(r/r_0)^{288}} \quad \text{and} \quad n_1 = \sum_N s(r) \tag{1}$$

with the ion-water distances r and the cutoff $r_0 = 0.3$ nm. With the hydration bias, all water molecules other than the five closest water molecules and the selected exchanging water are forced to remain outside the first hydration shell by setting

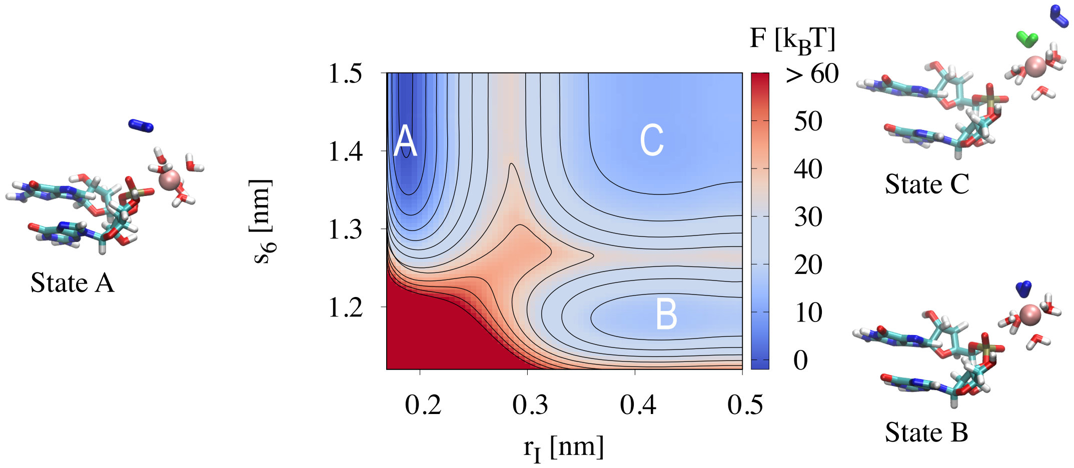

Figure S2: Two-dimensional free energy landscape $F(r_I, s_6)$ of the Mg^{2+}-O1P distance r_I and the hydration parameter s_6. s_6 is the sum of the distances to the five closest water molecules (shown in red and white) and the selected water molecule W_{ex} for which the biasing umbrella potential is applied (shown in blue). The green water molecule indicates one of the $N-6$ unlabeled water molecules. The energy contour spacing is 5 $k_\mathrm{B}\mathrm{T}$.

$n_1 = 0$ using a force constant of $k_h = 1000$ kJ/mol.

3 Dependence of the performance on the deep learning model architecture

The quality of the deep learning model strongly depends on the choice of the underlying model architecture. To illustrate the dependence of the accuracy on the deep learning model architecture, 12 architectures out of several thousands were used to predict the transition state ($0.45 < p_\mathrm{A}^\mathrm{pred} < 0.55$) and to calculate the resulting committor distribution $p(p_\mathrm{A}^\mathrm{true})$. The model architectures selected for this analysis were the following:
Optimal model: 5 hidden layers with 256 neurons, ReLU activation and an initial learning rate of $1.9 * 10^{-4}$. A dropout layer with 30% dropout is placed directly before the output layer.
Model 1: 5 hidden layers with 256 neurons, ReLU activation and an initial learning rate of $2.8 * 10^{-3}$. A dropout layer with 30% dropout is placed after the second hidden dense layer.
Model 2: 3 hidden layers with 192 neurons, ReLU activation and an initial learning rate of $1.6 * 10^{-3}$. A dropout layer with 20% dropout is placed after the input layer.
Model 3: 4 hidden layers with 192 neurons, ReLU activation and an initial learning rate of $3.8 * 10^{-3}$. A dropout layer with 20% dropout is placed after the input layer.
Model 4: 3 hidden layers with 192 neurons, ReLU activation and an initial learning rate of $5.9 * 10^{-3}$. A dropout layer with 10% dropout is placed after the input layer.
Model 5: 4 hidden layers with 224 neurons, ReLU activation and an initial learning rate of $1.3 * 10^{-4}$. A dropout layer with 30% dropout is placed after the input layer.
Model 6: 5 hidden layers with 256 neurons, ReLU activation and an initial learning rate of $1.3 * 10^{-3}$ without dropout layer.
Model 7: 5 hidden layers with 192 neurons, ReLU activation and an initial learning rate of $8.0 * 10^{-4}$. A dropout layer with 20% dropout is placed after the input layer.
Model 8: 4 hidden layers with 224 neurons, ReLU activation and an initial learning rate of $4.3 * 10^{-4}$. A dropout layer with 20% dropout is placed after the second hidden dense layer.
Model 9: 5 hidden layers with 256 neurons, SELU activation and an initial learning rate of $3.6 * 10^{-3}$ without dropout layer.
Model 10: 6 hidden layers with 256 neurons, ReLU activation and an initial learning rate of $4.0 * 10^{-3}$ without dropout layer.
Model 11: 3 hidden layers with 224 neurons, ReLU activation and an initial learning rate of $2.0 * 10^{-3}$ without dropout layer.
Model 12: 2 hidden layers with 192 neurons, ReLU activation and an initial learning rate of $5.3 * 10^{-3}$. A dropout layer with 30% dropout is placed after the first hidden dense layer.

4 Deep learning model with the two most important features

In order to determine the error one makes upon discarding most of the features, we performed additional calculations in which we used only the two most important features. Figure S3A, B shows the direct comparison of the prediction using all 83 features or for using only the two most relevant features. The results show, that with only two features the prediction is not very accurate (Figure S3B with RMS error of 23%). Further insight into the quality of the predictions with only two features is obtained from the distribution of the transition states (Figure S3C). With the two most important features the distribution is very broad and the quality of the prediction is compatible to the one by the human expert.

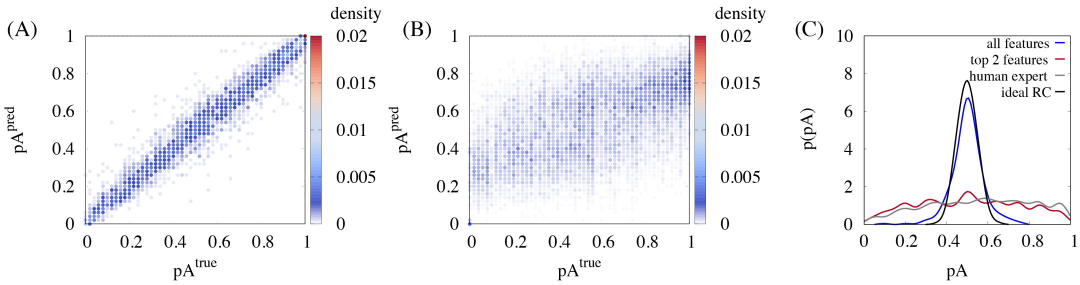

Figure S3: Committor values $p_{\mathrm{A}}^{\mathrm{pred}}$ predicted by the deep neural net using a different number of features correlated with the values $p_{\mathrm{A}}^{\mathrm{true}}$ obtained from the simulations. (A) With 83 features the RMS error of the prediction is 6.5%. (B) With the two most important features the RMS error is 23%. (C) Comparison of the committor distribution p($p_{\mathrm{A}}^{\mathrm{true}}$) for transition states selected from the deep learning algorithm using all features (blue) and using only the 2 most important features (red). For comparison, the ideal binomial distribution (black) and the result from the human expert (gray) are shown.

5 Influence of the splitting

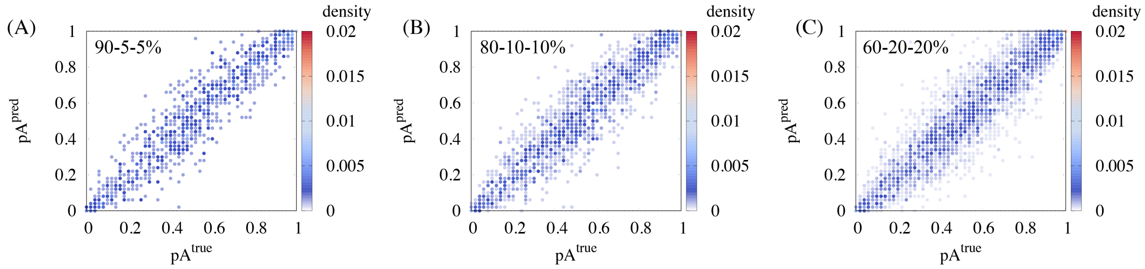

Figure S4: Influence of the splitting: Committor values $p_{\mathrm{A}}^{\mathrm{pred}}$ predicted by a deep neural net using different amounts of data for training, test and validation correlated with the values $p_{\mathrm{A}}^{\mathrm{true}}$ obtained from the committor simulations. (A) 90-5-5, (B) 80-10-10 and (C) 60-20-20%.

The influence of the splitting was analyzed by using the following amounts of data for training, test and validation: 90-5-5, 80-10-10 and 60-20-20%. We selected the optimal model by running a Keras Tuner random search in the space of model parameters (100 different model architectures) and training each model for 50 epochs. The results are shown in Figure S4. The RMS error of the prediction is 10.0% (90-5-5), 10.1% (80-10-10) and 10.6% (60-20-20). Larger amounts of data for training yield slightly better predictions as expected. Overall, the results are robust and the details of the splitting does not have a significant influence.

6 Commitment probability from constrained simulations

To test the influence of the second hydration shell on the commitment probability, we performed additional constrained simulations. For 600 shooting points, the positions of Mg^{2+}, RNA, five closest water molecules and exchanging water were kept constant. All other atoms were equilibrated for 1 ns in the NVT ensemble. Subsequently, 100 trajectories were initiated with velocities drawn from a Maxwell-Boltzmann velocity distribution and run forward and backward for 2 ps (see main manuscript for further details). The commitment probability p_{A} was calculated from the fraction of trajectories that reached state A. Figure S5 shows a comparison of the commitment probability for 600 conformations drawn directly from transition path sampling and for the same conformations after the constrained equilibration described above. Clearly, the values of

p_A are different for the unconstrained and the constrained conformations. We conclude that the positions of Mg^{2+}, RNA, five closest water molecules and exchanging water are insufficient to predict p_A. Consequently, the exchange reaction is influenced by water molecules outside the first hydration shell. A quantitative description should therefore include the water molecules from the second hydration shell or even beyond.

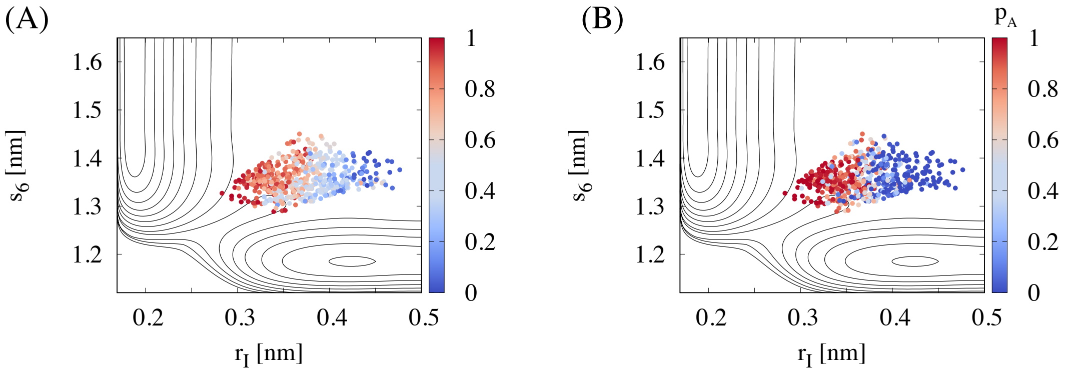

Figure S5: Committor probability for 600 shooting points as function of the features r_I and s_6. (A) Committor probability for conformations from transition path sampling. (B) Committor probability after a constrained equilibration in which the positions of Mg^{2+}, RNA, five closest water molecules and exchanging water were kept constant and the solvent was relaxed. The energy contour spacing is 5 k_BT.

7 Features

Table S1 lists all features used for deep learning. The features include all distances r_i between Mg^{2+} and the 20 closest water molecules, distances between Mg^{2+} and different RNA atoms and the Cl^- ion, all angles a_i formed between O1P, Mg^{2+} and the 20 closest water molecules, Steinhardt-Nelson order parameters, tetrahedral order parameters and numbers of hydrogen bonds for different groups of atoms. Hereby, the tetrahedral order parameter calculates the degree to which the water molecules around O1P or W_{ex} have a tetrahedral order. It is defined as

$$t_i = \frac{1}{\sum_j \sigma(r_{ij})} \sum_j \sigma(r_{ij}) \left[\frac{(x_{ij}+y_{ij}+z_{ij})^3}{r_{ij}^3} + \frac{(x_{ij}-y_{ij}-z_{ij})^3}{r_{ij}^3} + \frac{(-x_{ij}+y_{ij}-z_{ij})^3}{r_{ij}^3} + \frac{(-x_{ij}-y_{ij}+z_{ij})^3}{r_{ij}^3} \right] \tag{2}$$

where r_{ij} is the magnitude of the vector connecting atom i and atom j and x_{ij}, y_{ij} and z_{ij} are its three components. $\sigma(r_{ij})$ is a switching function with a cutoff of $r_0 = 0.35$ nm.

The Steinhardt-Nelson order parameters measure to which degree the hydration shell is ordered. The 3rd Steinhardt-Nelson order parameter around atom i is calculated from

$$q_{3i} = \sqrt{\sum_{m=-3}^{3} q_{3m}^*(i) q_{3m}(i)} \quad \text{with} \quad q_{3m}(i) = \frac{\sum_j \sigma Y_{3m}(\mathbf{r}_{ij})}{\sum_j \sigma} \tag{3}$$

where Y_{3m} is the 3rd order spherical harmonics and σ is a switching function. σ is one for the atoms of the hydration shell under consideration and zero otherwise.

The 4th Steinhardt-Nelson order parameter is calculated from

$$q_{4i} = \sqrt{\sum_{m=-4}^{4} q_{4m}^*(i) q_{4m}(i)} \quad \text{with} \quad q_{4m}(i) = \frac{\sum_j \sigma Y_{4m}(\mathbf{r}_{ij})}{\sum_j \sigma} \tag{4}$$

where Y_{4m} is the fourth order spherical harmonics.

The 6th Steinhardt-Nelson order parameter is calculated from

$$q_{6i} = \sqrt{\sum_{m=-6}^{6} q_{6m}^*(i) q_{6m}(i)} \quad \text{with} \quad q_{6m}(i) = \frac{\sum_j \sigma Y_{6m}(\mathbf{r}_{ij})}{\sum_j \sigma} \tag{5}$$

where Y_{6m} is the sixth order spherical harmonics. All distances, angles, Steinhardt-Nelson order parameters and tetrahedral order parameter were calculated with PLUMED. The number of hydrogen bonds were calculated with GROMACS.

Table S1: Features used for deep learning.

feature	definition
r_I	distance between Mg^{2+} and the phosphate oxygen O1P
$r_1 - r_5$	distances of the 5 closest water molecules
$r_{\rm ex}$	distance between Mg^{2+} and the exchanging water
$r_6 - r_{20}$	distances of the 6-20 closest water molecules
$a_1 - a_{20}$	angles between the oxygen atoms of the 20 closest waters, Mg^{2+} and O1P
Δr	$r_I - r_{ex}$
$r_{\rm Cl}$	Mg^{2+}-Cl^- distance
$r_{\rm O2P}$	Mg^{2+}-O2P distance
$r_{\rm P}$	Mg^{2+}-P distance
$\alpha_{\rm ex}$	angle O1P, Mg^{2+}, W_{ex}
s_6	Sum of distances for 5 closest waters + exchanging water
λ	$\mathrm{atan2}(s_6 - 1.18, r_I - 0.18)$
q_{i0}	i-th Steinhardt-Nelson order parameter (SNOP) for oxygen atoms of 5 closest waters (i=3, 4, 6)
q_{i1}	i-th SNOP for oxygen of 5 closest waters, O1P and oxygen atoms of W_{ex} (i=3, 4, 6)
q_{i2}	i-th SNOP for oxygen atoms of second hydration shell (i=3, 4, 6)
q_{i3}	i-th SNOP for oxygen atoms of second hydration shell, O1P and oxygen of W_{ex}
q_i	i-th SNOP for all oxygen atoms in first and second hydration shell and O1P
$q_{i0}^{\rm W}$	i-th SNOP for 5 closest waters including hydrogens (i=3, 4, 6)
$q_{i1}^{\rm W}$	i-th SNOP for 5 closest waters, O1P and W_{ex} (i=3, 4, 6)
$q_{i2}^{\rm W}$	i-th SNOP for water in second hydration shell (i=3, 4, 6)
$q_{i3}^{\rm W}$	i-th SNOP for water in second hydration shell, O1P and W_{ex}
$q_i^{\rm W}$	i-th SNOP for water in first and second hydration shell and O1P
$q_4^{\rm ex}$	4-th SNOP of water oxygens within 0.35 nm of W_{ex}
$q_4^{\rm O1P}$	4-th SNOP of water oxygens within 0.35 nm of O1P
$t_{\rm ex}$	tetrahedral symmetry of water within 0.35 nm of W_{ex}
$t_{\rm O1P}$	tetrahedral symmetry of water within 0.35 nm of O1P
$h_{\rm ex}^1$	h-bonds of W_{ex} and first hydration shell
$h_{\rm ex}^2$	h-bonds of W_{ex} and second hydration shell
$h_{\rm O1P}^1$	h-bonds between O1P and water from the first hydration shell
$h_{\rm O1P}^2$	h-bonds between O1P and water from the second hydration shell
$h_{\rm O2P}^1$	h-bonds between O2P and water from the first hydration shell
$h_{\rm O2P}^2$	h-bonds between O2P and water from the second hydration shell
h	h-bonds between first and second hydration shell
s_2	$s_2=\sum_{i=1}^{12} \exp(-\sigma((r_i - r_0)^2) + (a_i - a_0)^2)$ with $\sigma = 50$, $r_0 = 0.35$ nm, $a_0 = 120°$

Table S2: Feature ranking according to combined and single feature relevance and negative log likelihood loss l.

feature	l	c-rank	l	s-rank
$r_{\mathrm{I}} - r_{\mathrm{ex}}$	107.78	83	67.46	83
r_{I}	99.26	82	67.2	82
q_{40}	93.42	81	66.07	81
r_{P}	90.90	80	65.73	80
h^1_{OP}	89.73	79	65.22	72
h^1_{ex}	89.00	78	65.43	79
a_1	88.10	77	65.31	75
s_6	87.32	76	65.02	58
a_7	86.82	75	65.37	76
a_8	85.94	74	65.11	67
q_{41}	85.41	73	65.43	78
q^{W}_{40}	85.28	72	64.97	50
t_{O1P}	85.07	71	65.08	65
r_6	84.57	70	64.91	38
$\|r_{\mathrm{I}} - r_{\mathrm{ex}}\|$	83.90	69	65.12	69
h^2_{O1P}	83.84	68	65.24	73
r_{Cl}	83.17	67	65.42	77
q_{32}	83.16	66	64.97	52
h^2_{ex}	82.81	65	65.08	64
q_{60}	82.79	64	65.03	59
q^{ex}_4	82.39	63	65.21	71
q_3	82.14	62	65.05	60
r_7	81.70	61	65.1	66
q^{W}_{32}	81.56	60	64.92	41
h^2_{O2P}	81.30	59	64.96	48
q_{33}	80.82	58	65.28	74
a_9	80.60	57	64.92	42
q_{31}	80.23	56	64.93	46
r_{O2P}	80.04	55	65.05	61
r_{17}	79.67	54	65.07	63
$q6$	79.43	53	65.12	68
q^{W}_{62}	79.28	52	64.79	16
q^{O1P}_4	79.11	51	64.99	54
t_{ex}	78.83	50	65.17	70
q^{W}_4	78.48	49	64.89	36
q_{63}	78.16	48	64.99	55
r_{18}	77.88	47	64.98	53
r_{20}	77.56	46	64.96	49
q_4	77.37	45	64.93	45
q^{W}_6	76.95	44	65.01	57
r_9	76.74	43	64.83	24
a_2	76.54	42	64.92	39
λ	76.19	41	64.88	33
q_{62}	75.95	40	64.92	40
r_{16}	75.50	39	64.9	37
q^{W}_3	75.35	38	64.93	44
r_{ex}	74.88	37	64.87	32
q_{42}	74.55	36	64.97	51
s_2	74.35	35	65.06	62
q^{W}_{60}	74.12	34	65	56
q_{61}	73.82	33	64.93	47
q^{W}_{41}	73.39	32	64.93	43
q_{41}	73.12	31	64.85	26
r_{19}	72.80	30	64.88	35
a_{10}	72.42	29	64.86	30
r_{10}	72.13	28	64.81	18

feature	l-c	c-rank	l-s	s-rank
r_{14}	71.82	27	64.85	28
q_{63}^{W}	71.51	26	64.82	21
r_{15}	71.07	25	64.86	29
r_{12}	70.86	24	64.78	14
r_{8}	70.49	23	64.87	31
a_{11}	70.06	22	64.81	17
r_{11}	69.81	21	64.88	34
q_{61}^{W}	69.47	20	64.82	23
a_{6}	69.15	19	64.85	27
q_{30}^{W}	68.81	18	64.81	20
q_{43}^{W}	68.41	17	64.72	3
h_{O2P}^{1}	68.16	16	64.84	25
q_{30}	67.89	15	64.81	19
h	67.61	14	64.82	22
q_{42}^{W}	67.31	13	64.75	11
r_{5}	67.03	12	64.76	12
a_{3}	66.82	11	64.78	15
a_{12}	66.61	10	64.73	4
q_{31}^{W}	66.32	9	64.74	9
r_{13}	66.09	8	64.75	10
a_{5}	65.83	7	64.77	13
r_{1}	65.63	6	64.73	8
q_{33}^{W}	65.40	5	64.73	5
r_{2}	65.20	4	64.73	6
r_{4}	65.03	3	64.73	7
r_{3}	64.87	2	64.71	2
a_{4}	64.69	1	64.71	1
all	64.55	0	64.55	0
